# 左肺癌对侧乳腺转移男性患者1例

**DOI:** 10.3779/j.issn.1009-3419.2010.11.17

**Published:** 2010-11-20

**Authors:** 雁启 丁, 静 周, 红玲 单

**Affiliations:** 272200 金乡，济宁医学院附属金乡医院呼吸内科 Department of Respiratory Medicine, Jinxiang Hospital Affiliated to Jining Medical College, Jinxiang 272200, China

乳腺癌的肺部转移临床多见，而原发肺癌的乳腺转移较少见。济宁医学院附属金乡医院呼吸内科收治1例原发肺癌伴乳腺转移患者，现报道如下。

## 1 病例资料

患者，男，62岁，因“咳嗽、咯血1天”于2007年10月7日入院。患者于1天前无诱因出现阵发性剧烈咳嗽，咯鲜血，初为痰中带血，后为大口鲜血，约200 mL，无发热、胸痛，无憋喘、晕厥。既往有糖尿病，肝硬化病史4年。

入院查体：BP 160/80 mmHg，神志清，精神可，浅表淋巴结无肿大，双肺呼吸音清，未闻及啰音。右乳腺可触及一鸡蛋大小肿物，质硬，活动度差，轻压痛，边界尚清。心率88次∕分，心律齐，无杂音。腹软，肝脾肋下未及。辅助检查：白细胞6.0×10^9^/L，中性粒细胞占77.8%，总蛋白66.4 g/L，白蛋白37.6 g/L，白/球比值1.31，AST 42 U/L（参考值0 U/L-40 U/L），ALT 31 U/L（参考值0 U/L-40 U/L），空腹血糖8.6 mmol/L，癌胚抗原（carcinoembryonic antigen, CEA）10.7 ng/mL（参考值< 5.0 ng/mL）。肝胆胰脾彩超示慢性肝病，胆囊炎伴胆囊多发结石。右乳腺彩超示右腋下多发低回声（考虑增大淋巴结），右乳头上方低回声占位，大小约2.5 cm ×1.3 cm，边界尚清，内回声欠均匀。彩色多普勒（color doppler flow imaging, CDFI）：团块内及周边可见血流信号，其内探及动脉频谱，RI为0.9。胸部增强CT示左肺门见一软组织密度肿块，轻度强化，纵隔淋巴结无肿大，肺内无结节灶（图 1A，图 1B）。纤维支气管镜示左肺上叶支气管见一肿块，活检病理示鳞癌（图 2A）。行右乳腺肿物穿刺检查为血性少许物，镜检：可见大量异形腺上皮，可见较多核分裂，考虑右乳腺癌可能性大，建议术中冰冻切片。为明确诊断，转普外科行右乳腺癌改良根治术，术中冰冻切片病理示右乳腺癌，倾向于鳞癌。手术后病理示右乳腺鳞癌2级，癌肿大小3.3 cm ×3.0 cm×2.2 cm，腋下淋巴结未见转移（0/6），乳头、皮肤和标本基底切线未见累及（图 2B），免疫组化：雌激素受体（estrogen receptor, ER）（-），孕激素受体（progestogerone receptor, PR）（-），p53（+），高分子量细胞角蛋白（high molecular weight cytokeratin, CK-H）（+），低分子量细胞角蛋白（low molecular weight cytokeratin, CK-L）（+），考虑右乳腺转移鳞癌，来自肺的可能性大（图 3A，图 3B，图 3C）。

**1:**
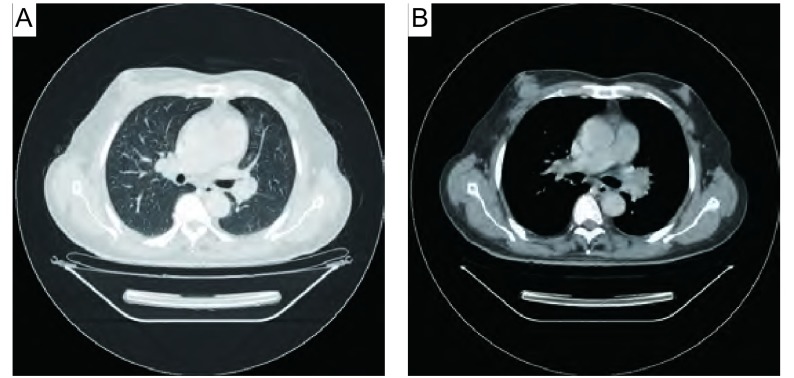
胸部CT。A：（平扫）肺窗示左肺门增大；B：（增强扫描）纵隔窗示左肺门见一软组织密度肿块，轻度强化。 Chest CT. A: (plain scan) lung window: Chest CT scan shows the left hilar increases; B: (enhanced scan) mediastinum window displays: There is a soft tissue density mass in the left hilar, and it is mild enhancement.

**2:**
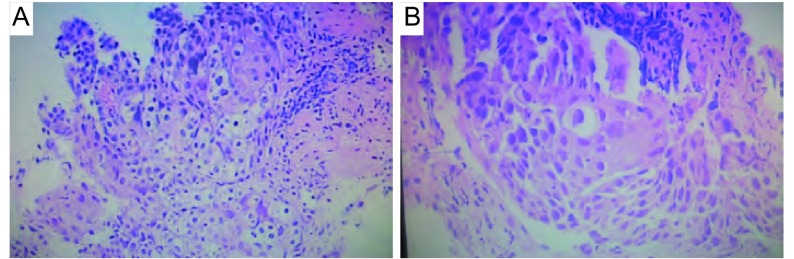
病理结果。A：（左肺上叶支气管活检）鳞状细胞癌（HE, ×100）；B：右乳腺转移鳞癌（HE, ×100）。 Pathological results. A: (left upper lobe bronchial biopsy) squamous cell carcinoma (HE, ×100); B: squamous cell carcinoma of right breast with metastasis (HE, ×100).

**3:**
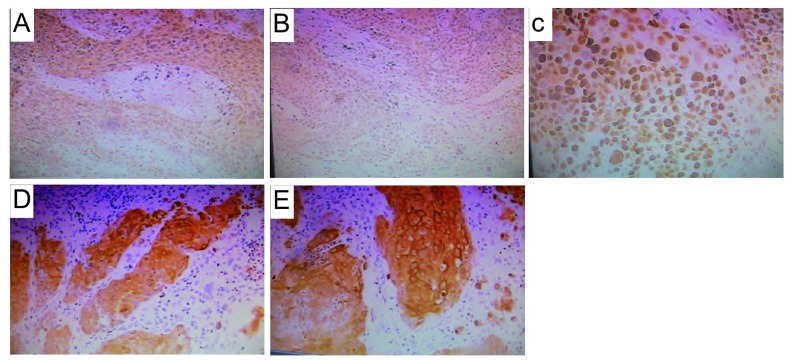
右乳腺转移鳞癌免疫组化结果。A：ER阴性（HE, ×100）；B：PR阴性（HE, ×100）；C：p53阳性（HE, ×100）；D：高分子量细胞角蛋白（CK-H）阳性（HE, ×100）；E：低分子量细胞角蛋白（CK-L）阳性（HE, ×100）。 Immunohistochemistry of squamous cell carcinoma of right breast with metastasis. A: ER negative (HE, ×100); B: PR negative (HE, ×100); C: P53 positive (HE, ×100); D: cytokeratin, high molecular weight (CK-H) positive (HE, ×100); E: cytokeratin, low molecular weight (CK-L) positive (HE, ×100).

## 2 讨论

临床上，肺癌是一种很常见的恶性肿瘤。肺癌常见的转移部位是肺内、脑及骨骼，但转移至对侧乳腺少见。乳腺转移性肿瘤比较少见，大约占乳腺恶性肿瘤的1%-5%。有报道^[1]^，中国医学科学院肿瘤医院收治的3 094例乳腺癌中，继发性乳腺癌仅7例（0.23%）。继发性乳腺癌多由对侧乳腺癌转移而来，而来自其它部位的恶性肿瘤较少见。男性乳腺癌罕见，仅占全部乳腺癌的1%，病理类型以浸润性导管癌最常见。一般来说，肺鳞癌倾向于表现为通过直接扩散侵犯局部邻近组织，与其它原发性肺癌组织类型相比，鳞癌较少发生远处器官转移，而乳腺转移性鳞癌更罕见。查阅中国知网CNKI期刊全文数据库（1994年-2009年），查到文献报道原发性肺癌伴乳腺转移癌12例，女9例，男3例，其中鳞癌仅2例^[1-11]^。Behranwala等^[12]^认为乳腺鳞癌是一种罕见的癌症类型。Rosen^[13]^亦认为乳腺鳞癌是一种罕见的肿瘤，所占比例不到乳腺癌的0.1%。陈蓉等^[9]^报道的1例男性患者，年龄89岁，为左肺上叶前段周围型肺癌并右侧乳腺、右腋窝淋巴结转移，因而认为对大龄乳腺癌患者，诊断时应考虑有继发性乳腺癌的可能性。

由于乳腺是性器官，所以正常乳腺均有ER、PR的表达。约60%-70%的原发乳腺癌中ER、PR为阳性，肺原发肿瘤ER、PR均为阴性。

有报道，Raab可区分原发肺癌及乳腺癌肺转移，应用大囊液蛋白-15、ER、S-100蛋白免疫组化检测，如以上指标均为阳性，肺部肿瘤为转移；若以上指标为阴性，而CEA（+），则肺部肿瘤为原发^[11]^。绝大多数的鳞状细胞癌可表现CK-H、细胞角蛋白5/6和CEA的高表达。CK是上皮细胞特征性标记物。该患者免疫组化ER、PR均为阴性，而p53、CK-H、CK-L为阳性。结合病理及免疫组化，可以明确诊断为左肺鳞癌并对侧乳腺转移癌。分析该患者出现乳腺转移的原因有：①通过胸内淋巴转移至右侧乳腺；②肿瘤细胞沿淋巴循环经胸导管进入静脉，经体循环到达对侧乳腺；③肿瘤细胞直接进入血液循环，引起远处转移^[7]^。

原发性肺癌发生于支气管粘膜上皮，常有刺激性咳嗽、胸闷、胸痛、痰中带血等症状。乳腺癌主要症状有：乳腺肿块及疼痛、乳头溢液及乳头改变、皮肤改变、腋窝淋巴结肿大等。乳腺癌的肺部转移常见，常表现为单侧或双侧肺野结节性多发转移，病灶大小不一，形状多为圆形或类圆形，无分叶或毛刺，密度较淡，边界清晰。乳腺癌一般极少转移至支气管，而且早期很少有咳嗽等刺激症状。宋道前等^[14]^报告76例肺转移瘤患者，无一例出现支气管内转移。肺癌转移至乳腺也很少见。原发性乳腺癌与继发性乳腺癌的治疗与预后不同，因此鉴别诊断有很重要的意义。鉴别诊断主要依靠临床及病理检查，结合肿瘤的部位（如支气管、肺间质、乳腺导管等）应该有助于鉴别。

由此看来，虽然乳腺并非常见的恶性肿瘤转移部位，乳腺也确实是肺癌转移的靶器官之一，这使我们在今后的工作中对本病有了进一步的认识。
